# An ancient retroviral RNA element hidden in mammalian genomes and its involvement in co-opted retroviral gene regulation

**DOI:** 10.1186/s12977-021-00580-2

**Published:** 2021-11-10

**Authors:** Koichi Kitao, So Nakagawa, Takayuki Miyazawa

**Affiliations:** 1 Laboratory of Virus-Host Coevolution, Institute for Frontier Life and Medical Sciences, Kyoto University, 53 Shogoin-Kawaharacho, Sakyo-ku, Kyoto, 606-8507 Japan; 2 Department of Molecular Life Science, Tokai University School of Medicine, Isehara, Kanagawa 259-1193 Japan

**Keywords:** Endogenous retroviruses, RNA regulatory element, *Syncytin*, Mammalian genomes

## Abstract

**Background:**

Retroviruses utilize multiple unique RNA elements to control RNA processing and translation. However, it is unclear what functional RNA elements are present in endogenous retroviruses (ERVs). Gene co-option from ERVs sometimes entails the conservation of viral *cis*-elements required for gene expression, which might reveal the RNA regulation in ERVs.

**Results:**

Here, we characterized an RNA element found in ERVs consisting of three specific sequence motifs, called SPRE. The SPRE-like elements were found in different ERV families but not in any exogenous viral sequences examined. We observed more than a thousand of copies of the SPRE-like elements in several mammalian genomes; in human and marmoset genomes, they overlapped with lineage-specific ERVs. SPRE was originally found in human *syncytin-1* and *syncytin-2*. Indeed, several mammalian *syncytin* genes: mac-syncytin-3 of macaque, syncytin-Ten1 of tenrec, and syncytin-Car1 of Carnivora, contained the SPRE-like elements. A reporter assay revealed that the enhancement of gene expression by SPRE depended on the reporter genes. Mutation of SPRE impaired the wild-type *syncytin-2* expression while the same mutation did not affect codon-optimized *syncytin-2*, suggesting that SPRE activity depends on the coding sequence.

**Conclusions:**

These results indicate multiple independent invasions of various mammalian genomes by retroviruses harboring SPRE-like elements. Functional SPRE-like elements are found in several *syncytin* genes derived from these retroviruses. This element may facilitate the expression of viral genes, which were suppressed due to inefficient codon frequency or repressive elements within the coding sequences. These findings provide new insights into the long-term evolution of RNA elements and molecular mechanisms of gene expression in retroviruses.

**Supplementary Information:**

The online version contains supplementary material available at 10.1186/s12977-021-00580-2.

## Background

Just as the traces of ancient organisms remain as fossils, traces of ancient retroviruses remain as DNA sequences in host genomes. They are called endogenous retroviruses (ERVs), remnants of ancient retroviruses incorporated into the genome through infection of host germ cells. ERVs are not mere fossil records, as some are still active as protein-coding genes or regulatory elements in the host genome. Their functional features have been inherited from ancestral viruses: for example, the placental fusogenic Syncytin proteins from the fusogenic envelope protein [1], and lineage-specific host enhancers/promoters from the long terminal repeat (LTR) of viral multifunctional regulatory elements [2]. On the other hand, much is unknown about the role of RNA elements in ERVs. Recent studies reported that several host RNA-binding proteins interact with ERV transcripts [3]. However, it is unclear what kinds of unique RNA elements are present in ERVs and their biological significance for the hosts.

RNA elements provide a layer of post-transcriptional regulation to balance the gene expression of the viral proteins. Retroviruses have three genes: *gag* gene encoding the major structural protein; *pol* gene encoding RNase H, reverse transcriptase, and integrase; and *env* gene encoding envelope protein. Some retroviruses have additional RNA-binding proteins involved in post-transcriptional regulation. For example, human immunodeficiency virus (HIV)-1 belonging to the Genus *Lentivirus* encodes the regulatory protein termed Rev that binds to the Rev-responsive element (RRE) in the *env* region [4, 5]. The binding of Rev to RRE facilitates the export of un-spliced viral RNA to the cytoplasm with the host factor CRM1/XPO1 [6] as well as the translation of Env and regulatory and accessory proteins [7]. Similarly, Rex of human T-lymphotropic leukemia virus 1 belonging to the Genus *Deltaretrovirus* [8] and Rem of murine mammary tumor virus belonging to the Genus *Betaretrovirus* [9] are regulatory proteins that bind to their viral RNAs, allowing efficient viral replication. Mason-Pfizer monkey virus (MPMV), belonging to the Genus *Betaretrovirus*, does not have regulatory proteins but has an RNA element called the constitutive transport element (CTE). Bray et al. [10] initially reported that CTE could compensate for Rev-deficient HIV-1 replication. Then, it was revealed that the binding of the host protein TAP/NXF1 to CTE promotes nuclear transport and the translation of un-spliced viral RNA [11, 12]. Similarly, binging of NXF1 to the cytoplasmic accumulation element (CAE) in murine leukemia viral RNA belonging to the Genus *Gammaretrovirus* also promotes the expression of viral proteins [13]. Recent comprehensive mutagenesis approaches revealed that HIV-1 transcripts contain many undefined RNA elements required for efficient viral replication [14]. Thus, retroviruses have complex RNA elements in their short genomes that allow them to replicate efficiently.

Identification of such RNA elements from ERVs is challenging because accumulated mutations may have disrupted such elements. Exceptionally, HERV-K, which is a young ERV family, retains intact viral ORFs and shows polymorphic loci in the human genome [15, 16]. The post-transcriptional roles of its RNA-binding regulatory protein, Rec, and its binding RNA element have been demonstrated [17, 18]. Co-opted viral genes may provide important clues to investigate ancient viral RNA elements, given that these elements might have been similarly conserved to regulate the expression of co-opted genes. *Syncytin-1* is an *env* gene of ERVWE1 and contributes to cell fusion to differentiate multinucleated syncytiotrophoblasts in the human placenta [19, 20]. We previously reported that an RNA element located in the 3′ end of the protein-coding sequence and 3′ untranslated region (3′ UTR) of human *syncytin-1* is important for its protein expression and was named *syncytin* post-transcriptional regulatory element (SPRE) [21]. Indeed, human *syncytin-2*, another *syncytin* gene derived from an *env* gene of ERVFRDE1 [22], also contains a functional element in their 3′ UTR [21], although we have not examined it in detail. Such RNA elements would enable us to examine the RNA regulatory mechanisms of ancient retroviruses.

 In this study, a hidden Markov model (HMM)-based sequence search in an ERV database revealed the core motifs of SPRE. We found that the defined SPRE-like elements were widespread in 378 distinct ERV families but not in extant viruses. We also detected the SPRE-like elements in three non-human *syncytin* genes. A reporter assay verified their functionality and revealed the unique features allowing the protein-coding sequence of the target gene to affect the SPRE activity. These results provide new insights into ancient retroviral post-transcriptional regulation as well as its involvement in the co-opted genes from ERVs.

## Results

### The SPRE-core motif is functionally essential for SPRE activity

**Fig. 1:**
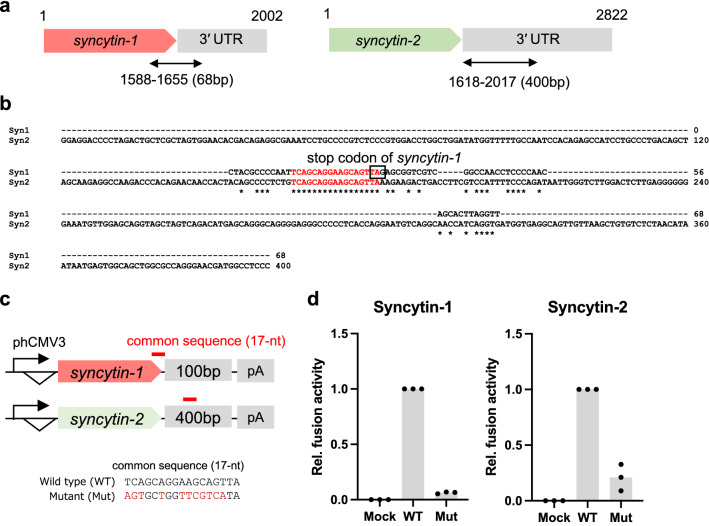
Identification of the 17-nt SPRE-core motif in *syncytin-1* and *syncytin-2*. **a** Locations of SPRE sequences, which increase protein expression of HIV-1 Gag in human *syncytin-1* and *syncytin-2* (68-nt and 400-nt, respectively). **b** The alignment of the two sequences. The 17-nt common sequence is represented in red. **c** Schematic representation of Syncytin-1 and Syncytin-2 expression plasmids and sequences of SPRE mutants. **d** Fusion-dependent luciferase assay in 293T cells transfected with Syncytin-1 and Syncytin-2 expression plasmids. WT; wild-type sequence, Mut; SPRE mutant. Each value was normalized by the luminous intensity of WT. Individual data are indicated as points, and medians are indicated as bars

Previously, we reported that a partial sequence in the 3′ end of the protein-coding sequence and subsequent 3′ UTR of human *syncytin-1* (68-nt) and a partial sequence in 3′ UTR of *syncytin-2* (400-nt) increase protein expression when inserted into 3′ UTR of an HIV-1 Gag expression plasmid [21] (Fig. 1a). We hypothesized that these sequences share functional RNA motifs. To explore the essential motif(s), two regulatory sequences were aligned and compared. We revealed that a 17-nt common sequence (5′-TCAGCAGGAAGCAGTTA-3′) is shared in *syncytin-1* and *syncytin-2* (Fig. 1b). Next, we examined whether the common sequence is essential for the expression of Syncytin-1 and Syncytin-2. We generated expression plasmids by cloning *syncytin-1* and *syncytin-2* with their 3′ UTRs and introduced mutations (11 nucleotides) into the 17-nt common sequence (Fig. 1c). Since this common sequence overlaps with the *syncytin-1* coding sequence, we generated mutants avoiding any amino acid substitutions in Syncytin-1. Syncytin expression levels were evaluated by a cell fusion-dependent luciferase assay utilizing the property that both Syncytin-1 and Syncytin-2 induce strong cell fusion. As a result, mutations in the common sequence markedly reduced cell fusion activities for Syncytin-1 and Syncytin-2 (Fig. 1d). Based on these results, we named the common sequence the ‘SPRE-core motif’, which is functionally essential for SPRE activity.

### SPRE-like elements in ERV families

**Fig. 2:**
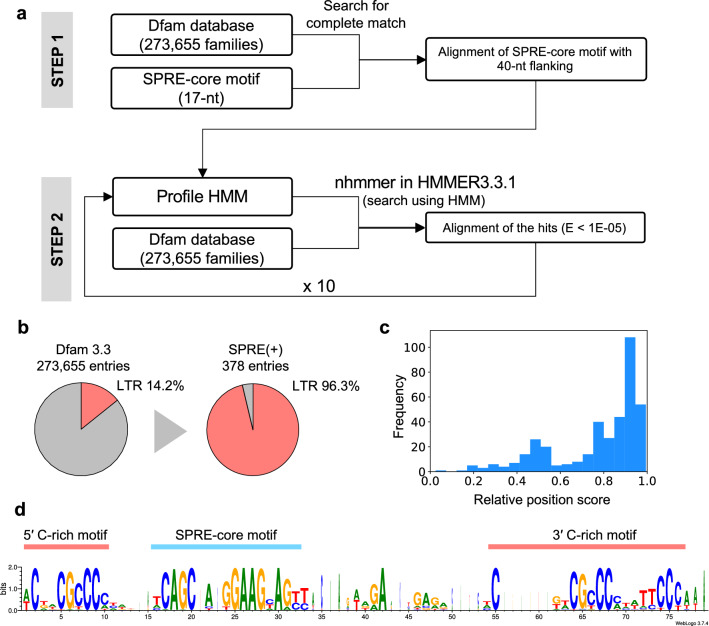
Sequence search for SPRE-like elements in ERV families. **a** Schematic representation of the two-step search for SPRE in the Dfam database. **b** Percentage of LTR-type retrotransposons (i.e., ERVs) among all repetitive DNA elements and ones with SPRE-like elements in the Dfam database. **c** A histogram of relative position scores of SPRE-like elements in Dfam families by the second-step search. The scores were calculated as [(S + E)/2]/L, where S and E are the start and end positions of the SPRE-like elements from the 5′ terminus, respectively, and L is the length of each Dfam family. Strandness was considered. **d** Sequence alignment and the sequence logo of 393 of SPRE-like elements. SPRE-core motif and C-rich motifs are indicated

Because *syncytin-1* and *syncytin-2* shared SPRE despite their different origins of ancestral viruses, we considered that more ERVs harbor SPRE-like elements. To test this, we searched for the SPRE-core motif against Dfam release 3.3, an open collection of 273,655 repetitive DNA families, including ERVs found in eukaryote genomes [23]. It is challenging to search the SPRE-like elements using such a short 17-nt motif while avoiding non-specific hits. Therefore, we adopted a two-step search strategy (Fig. 2a). In the first step, we extracted repetitive DNA families with a complete match to the SPRE-core motif from all Dfam entries and obtained 22 families harboring the SPRE-core motif. In the second step, SPRE-core motifs with 40-nt flanking sequences of both 5′ and 3′ sides of the 22 families were extracted and aligned (Additional file 1: Fig. S1). Then, we constructed a profile hidden Markov model (HMM) based on the alignment, and as step 2, we performed a sequence search using the nhmmer program of HMMER 3.3.1 [24]. The resultant hits were aligned and used to construct a new profile HMM. Then, the profile was subjected to re-searching by nhmmer. We repeated this process ten times as a trial and observed that the numbers of the hits peaked in the fourth cycle (Additional file 2: Fig. S2). We aimed to identify as many candidates of SPRE as possible, and therefore adopted the results of the fourth cycle, in which 393 hits from 378 families were obtained (Additional file 6: Table S1). All 378 families were repetitive DNA elements in mammalian genomes, and 96.3% of them were LTR-type retrotransposons (i.e., ERVs) (Fig. 2b and Additional file 6: Table S1). The positions of SPRE-like elements in the families were highly biased toward 3′-terminal regions, consistent with the fact that SPREs are found in 3′ UTR of *syncytin* genes (Fig. 2c). The sequence alignment revealed that there were two C-rich motifs in both 5′ and 3′ flanking regions of the SPRE-core motif (Fig. 2d), indicating that SPRE consists of three motifs: the SPRE-core motif and two C-rich motifs.

### No exogenous viruses with the SPRE-like elements and a number of the elements in host genomes

The profile HMM created by the above procedure allowed us to search for the SPRE-like elements in other sequence databases. To investigate whether currently prevailing (i.e., exogenous) viral genomes contain the SPRE-like elements, we searched for viral sequences obtained from all viral nucleotide sequences deposited in the NCBI virus database (https://www.ncbi.nlm.nih.gov/labs/virus/) using the nhmmer program with the HMM profile. We found no exogenous viruses that contain the SPRE-like elements (E-value < 1E−5). It should be noted that we obtained a hit with a relatively low E-value (0.0029); however, it was the multiple sclerosis-associated retrovirus (MSRV, GenBank Accession Number: AF127229), which likely arose from recombination of ERV-W-related sequences in the genome [25]. Therefore, MSRV cannot be considered an exogenous retrovirus.

**Fig. 3:**
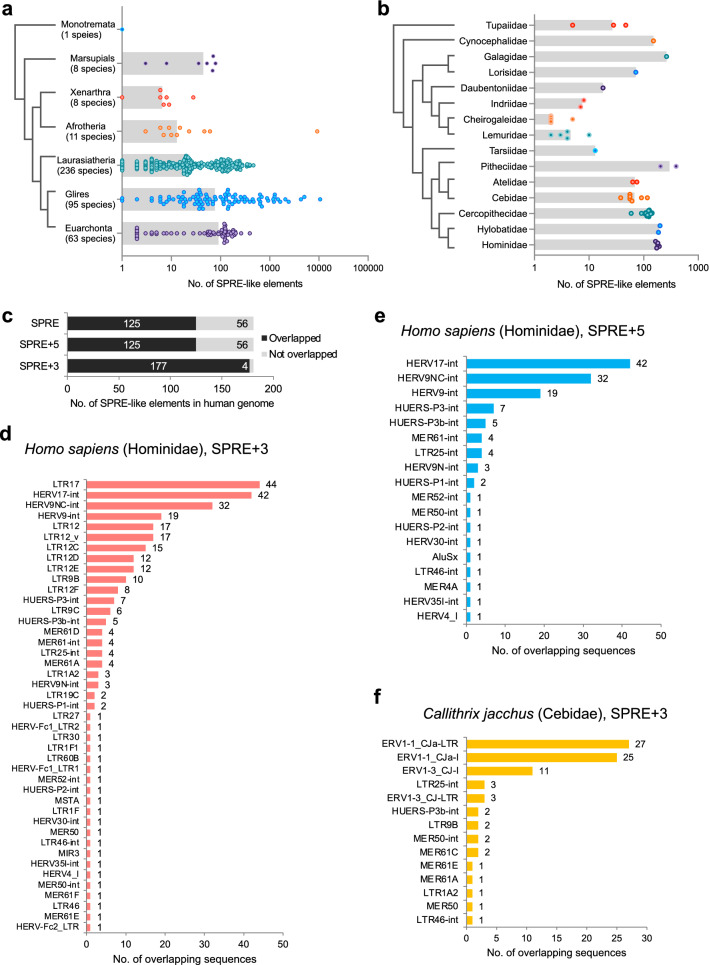
SPRE-like elements in host genomes. **a** Numbers of SPRE-like elements in host genomes. Each dot indicates the numbers of SPRE-like elements, and bar plots show the medians. **b** Numbers of SPRE-like elements in host families of Euarchonta. **c** The number of SPRE-like elements overlapping with RepeatMasker tracks in the human genome. SPRE-like elements by extending 50-nt sequences in the 5′ direction (SPRE+5) and in the 3′ direction (SPRE+3) are also examined. **d** The number of RepeatMasker tracks overlapping with SPRE+3 are indicated as bar plots. The “-int” of “_I” in repeat names indicates internal regions of ERVs. **e** The number of RepeatMasker tracks overlapping with SPRE+5 are indicated as bar plots. **f** The number of RepeatMasker tracks overlapping with SPRE+3 in the marmoset genome are indicated as bar plots

Next, we searched for the SPRE-like elements in genomes of 422 species of mammals and 499 species of birds available in the NCBI Assembly data (https://www.ncbi.nlm.nih.gov/assembly/). The SPRE-like elements were found in most mammalian genomes, and the numbers of hits varied markedly among species within the same clades (Fig. 3a and Additional file 7: Table S2). To investigate the distribution of the numbers of SPRE-like elements at the family level, we focused on Euarchonta, as an example, and found dynamic changes in the numbers among families (Fig. 3b). In contrast, in avian genomes, only five hits from four species were detected (Additional file 8: Table S3). It should be noted that we could not rule out the possibility of DNA contamination [26] for the following reasons: three out of five hits were located within short contigs (< 500 bp), and four out of five hits showed high similarity to sequences derived from *Homo sapiens* or *Mus musculus.* Hits in the genomes of Maroon-bellied parakeet (*Pyrrhura frontalis*) were significantly similar to *Oryzomys palustris* endogenous virus [27] and did not show any similarity to either *Homo sapiens* or *Mus musculus* sequences; however, a contig including this hit is also relatively short (GenBank ID: JAAAKN010044472.1, 1959-nt), and, therefore, the result was not still reliable. The risk of contamination in genomic assemblies also applies to the analysis of mammalian genomes.

To check whether the SPRE-like elements in mammalian genomes were derived from ERVs, the overlap of the SPRE-like elements with the repetitive DNA tracks from RepeatMasker (https://www.repeatmasker.org/) in genomes were analyzed (Additional file 9: Table S4). In the human genome, 125 out of the 181 SPRE-like elements overlapped with the RepeatMasker tracks (Fig. 3c). We also examined the overlap of SPRE-like elements by extending 50-nt sequences in both the 5′ and 3′ directions. The extension in the 5′ direction did not change the number of overlaps, whereas the extension in the 3′ direction results in 98% (i.e. 177 out of 181) elements found to overlap with RepeatMasker tracks (Fig. 3c). Therefore, most SPRE-like elements were derived from repeat elements. We also found that SPRE-like elements extended in the 3′ direction overlapped with LTRs and/or their internal regions (Fig. 3d). On the other hand, SPRE-like element extended in the 5′ direction did not overlap with LTRs (Fig. 3e). These data suggest that SPRE-like elements are in the internal regions and/or just upstream of LTRs. Especially, HERV17 (LTR17 and HERV17-int) and HERV9 (LTR12 and HERV9-int) overlapped with SPRE-like elements in the human genome (Fig. 3d and e). HERV17 and HERV9 were classified as the same supergroup related to ERV-W [28, 29]. These families were specific to Catarrhini, which is a clade including Hominidae, Hylobatidae, and Cercopithecidae. In marmoset (*Callithrix jacchus*) of Cebidae, 51 out of the 56 SPRE-like elements extended in the 3′ direction overlapped with RepeatMasker tracks, and most of them were ERV1-1_CJa-I and ERV1-3_CJ-I (Fig. 3f). These two families were specific to New World monkey, and ERV1-1_CJa-I was similar to ERV-W [30, 31]. Together, while SPRE-like elements are not present in the exogenous retroviruses, infections and/or transpositions of the different SPRE-harboring retroviruses independently pushed up the SPRE-like elements’ numbers in mammalian genomes.

### Various mammalian *syncytin* genes retain the SPRE-like elements

**Fig. 4:**
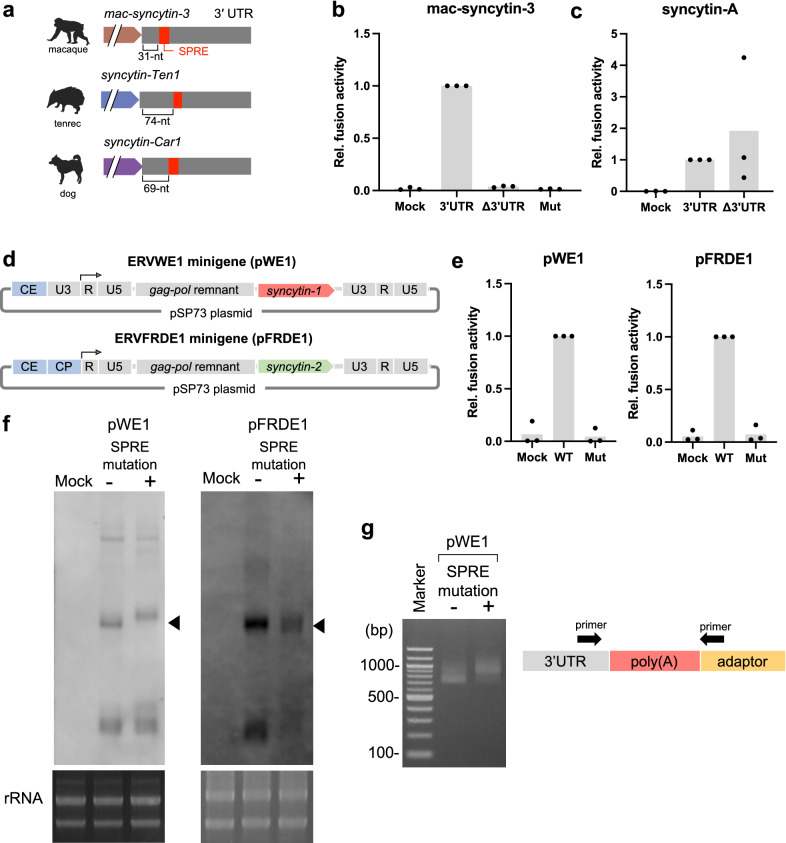
Identification of SPRE-like elements in different mammalian *syncytin* genes. **a** Schematic representation of SPRE-like elements in *mac-syncytin-3*, *syncytin-Ten1*, and *syncytin-Car1*. **b**,** c** Fusion-dependent luciferase assay in mac-Syncytin-3 and Syncytin-A using 293T cells. Each *syncytin* gene was cloned into the phCMV3 plasmid with or without 3′ UTR. A SPRE-core motif mutation similar to Fig. 2a was introduced into *mac-syncytin-3*. **d** Schematic representation of minigene plasmids of *syncytin-1*; pWE1 and *syncytin-2*; pFRDE1. The CMV enhancer and promoter were introduced upstream of the genes to enhance gene expression. CE; CMV enhancer, CP; CMV promoter. **e** Fusion-dependent luciferase assay of 293T cells transfected with pWE1 and pFRDE1. **f** Northern blot analysis in transcripts from 293T cells transfected with pWE1 and pFRDE1 minigenes with or without SPRE mutation. The arrowheads indicate the mRNA. **g** Poly(A) tail-targeted PCR in pWE1 transcripts

Next, we attempted to determine whether the SPRE-like elements are involved in the co-option of ERV-derived genes other than *syncytin-1* and *syncytin-2*. We examined other mammalian *syncytin* genes independently acquired from various distinct ERVs [32, 33]. We conducted an HMM search for the SPRE-like elements in protein-coding sequences and 1000-nt of 3′ flanking sequences of *mac-syncytin-3* in macaque [34], *syncytin-A*, and *-B* in mouse [35], *syncytin-Mar1* in squirrel [36], *syncytin-Ory1* in rabbit [37], *syncytin-Rum1* [38] and *fematrin-1* in cow [39], *syncytin-Car1* in dog [40], *syncytin-Ten1* in tenrec [41], and *syncytin-Opo1* in opossum [42]. As a result, we found the SPRE-like elements in the 3′ UTRs of *mac-syncytin-3*, *syncytin-Ten1*, and *syncytin-Car1* (Fig. 4a). In *mac-syncytin-3*, cell fusion activity was observed with the addition of 3′ UTR of *mac-syncytin-3*, but no cell fusion activity was observed without 3′ UTR or with a mutation in the SPRE-core motif (Fig. 4b). We also evaluated the function of 3′ UTR in the *syncytin* genes where the SPRE-like elements are not found using *syncytin-A* in mouse. It was revealed that Syncytin-A caused cell fusion irrespective of the presence of 3′ UTR, suggesting that *syncytin-A* does not have crucial RNA elements in 3′ UTR (Fig. 4c). The *syncytin* genes containing the SPRE-like elements are not phylogenetically related, as illustrated by a phylogenetic tree (Additional file 3: Fig. S3).

To infer the importance of SPREs in native mRNA forms of syncytin genes, which may include various intrinsic *cis*-acting elements affecting SPRE activity, we generated proviral minigene plasmids of *syncytin-1* and *syncytin-2*, including their 5′ and 3′ LTRs (Fig. 4d). Then, we introduced mutations into the SPRE-core motif and compared their protein expression levels by performing a cell fusion-dependent luciferase assay. As a result, we found that the mutations impaired the cell fusion activity (Fig. 4e). To examine the effects of SPRE mutations on the mRNA amounts and sizes, we performed Northern blot analysis using 293T cells transfected with *syncytin-1* and *syncytin-2* minigenes. As a result, the amounts of transcripts slightly decreased by the SPRE mutation in WT-*syncytin-2*, and no significant changes in the size of transcripts were observed (Fig. 4f). In *syncytin-1*, the amounts of transcripts were also slightly decreased, but the transcript’s size was increased by the SPRE mutation (Fig. 4f). We hypothesized that this band-shift was due to the longer length of the poly(A) tail. We conducted a poly(A) tail-targeted PCR and revealed that the SPRE mutant showed a longer poly(A) tail in *syncytin-1* mRNA (Fig. 4g). Several studies reported that highly expressed and well-translated transcripts have short poly(A) tails, probably because the poly(A) tails are decomposed in combination with the active translation and are bound by a minimal number of poly(A) binding proteins [43]. Therefore, the longer poly(A) tail produced by the SPRE mutation may suggest a reduced translation efficiency of *syncytin-1* mRNA.

### Functional analysis of the SPRE-like elements by the reporter assay

**Fig. 5:**
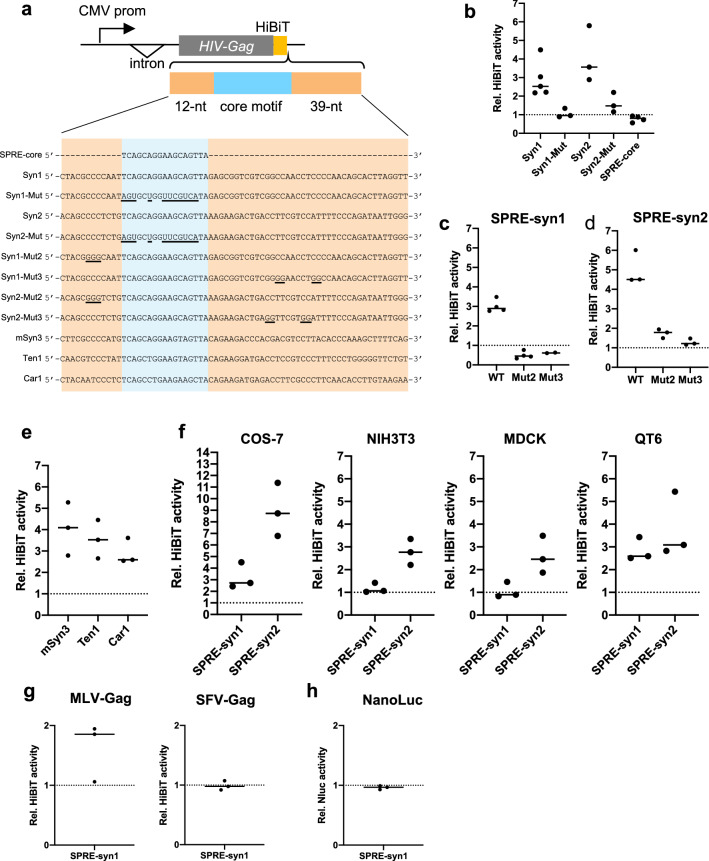
Functional analysis of SPRE-like elements using a reporter assay. **a** Schematic representation of the HIV-1 Gag-HiBiT (HG-HiBiT) reporter plasmid. Introduced mutations are indicated by underlining. **b** Luminous intensities were detected in 293T cells transfected with HG-HiBiT reporter plasmids. Each value was normalized by the luminous intensity from the HG-HiBiT expression plasmid without any additional sequences in 3′ UTR. **c**,** d** Functional investigation of C-to-G mutants of SPRE-syn1 and SPRE-syn2. **e** Functional investigation of SPRE-like elements of *mac-syncytin-3*; mSyn3, *syncytin-Ten1*; Ten1, and *syncytin-Car1*; Car1 by the HG-HiBiT reporter assay in 293T cells. **f** Functional investigation of SPRE-syn1 and SPRE-syn2 in COS7 (African green monkey), NIH3T3 (mouse), MDCK (dog), and QT6 (quail) by the HG-HiBiT reporter assay. Each value was normalized by the luminous intensity from the HG-HiBiT expression plasmid without any additional sequences in 3′ UTR in each cell line. **g** Functional investigation of SPRE activities in MLV-Gag and SFV-Gag. Each value was normalized by the luminous intensity of the respective reporter without any additional sequences in 3′ UTR. **h** Functional investigation of SPRE activities in NanoLuc

Next, we verified the functional activities of the SPRE-like elements using a reporter assay with HIV-1 Gag as a reporter protein. Since SPRE of *syncytin-1* (SPRE-syn1) was identified as a 68-nt sequence including the SPRE-core motif (17-nt) with 5′-flanking (12-nt) and 3′-flanking (39-nt) sequences to enhance HIV-1 Gag expression [21], we also constructed a reporter plasmid containing SPRE of *syncytin-2* (SPRE-syn2) in the same manner (Fig. 5a). We applied a recently developed luciferase system called HiBiT (Promega) to quantify the protein amounts by measuring the luminous activities. HIV-1 Gag was fused with the C-terminal HiBiT tag (HG-HiBiT), and SPRE-syn1 and SPRE-syn2 were then inserted into 3′ UTR of HG-HiBiT. As expected, the expression of HG-HiBiT was increased by insertion of the two SPREs, whereas the mutation introduced into the SPRE-core motif abolished the effects (Fig. 5b). We also found that the SPRE-core motif alone did not increase the protein expression level (Fig. 5b). To verify the importance of the C-rich motifs shown in Fig. 2d, we constructed C-to-G mutants of the C-rich motifs in SPRE-syn1 and SPRE-syn2 (Fig. 5a). These mutations impaired the enhancement of gene expression in both SPRE-syn1 and SPRE-syn2, suggesting that the C-rich motifs are crucial to SPRE activity (Fig. 5c and d). To test the functional activities of SPRE-like elements found in other mammalian *syncytin* genes (Fig. 4a), SPRE-like elements from *mac-syncytin-3*, *syncytin-Ten1*, and *syncytin-Car1* were inserted into HG-HiBiT (Fig. 5a). We found that all three SPRE-like elements enhanced the protein expression of HG-HiBiT (Fig. 5e).

Next, we tested the SPRE activity in mammalian and avian cell lines other than human cells (293T). We conducted the HG-HiBiT reporter assay in African green monkey (COS-7), mouse (NIH3T3), dog (MDCK), and quail (QT6) cell lines. Although SPRE-syn1 did not increase HG-HiBiT expression in NIH3T3 and MDCK, SPRE-syn2 was functionally active in all cell lines examined in this study (Fig. 5f). These results indicate that SPRE is functional in a wide range of host species.

Considering the fact that the SPRE-like elements were found in various ERVs and *syncytin* genes, they may enhance a wide variety of reporter genes not limited to HIV-1 Gag. To verify this hypothesis, SPRE-syn1 was inserted into 3′ UTR of Gag proteins of murine leukemia virus (MLV) and simian foamy virus (SFV) with HiBiT, and we measured their protein expression levels by HiBiT luciferase activity. Unexpectedly, the levels of gene expression enhancement by SPRE-syn1 were different among reporter genes: weak enhancement in MLV and no effect in SFV (Fig. 5g). SPRE-syn1 was also added downstream of NanoLuc as a non-viral control, but SPRE-syn1 did not affect the NanoLuc expression (Fig. 5h). These data suggest that SPRE activity is dependent on the reporter genes.

### SPRE activity depends on the protein-coding sequences

**Fig. 6:**
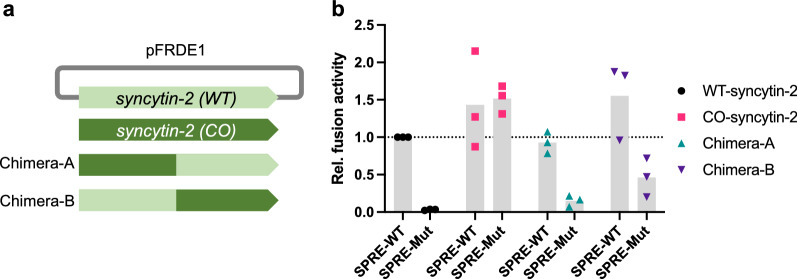
SPRE activity depends on the protein-coding sequence. **a** Schematic representation of codon-optimized (CO) *syncytin-2* and chimera *syncytin-2* expression plasmids. The wild-type (WT) *syncytin-2* in pFRDE1 indicated in Fig. 1e was replaced by CO-*syncytin-2*. **b** Fusion-dependent luciferase assay in WT-*syncytin-2*, CO-*syncytin-2*, Chimera-A, and Chimera-B, with wild-type SPRE (SPRE-WT) or mutated SPRE the same as Fig. 1c (SPRE-Mut). Each value was normalized by WT-*syncytin-2* with SPRE-WT, individual data points are indicated as points, and medians are indicated as bars

We hypothesized that SPRE activity depends on the protein-coding sequences. To test this, we modified the nucleotide sequence of the *syncytin-2* coding region without any amino acid changes by codon optimization. The coding sequence of the *syncytin-2* minigene described in Fig. 4d was modified as follows: the wild-type (WT), codon-optimized (CO), and two chimeric sequences of WT and CO (Chimera-A and -B) (Fig. 6a). *Syncytin-1* was not used in this analysis because its SPRE resides in the coding sequence. Fusion-dependent luciferase assays revealed that the gene expression of CO-*syncytin-2* was not affected by the mutation in the SPRE-core motif. We then examined whether this is caused by a specific region in the coding sequence of WT-*syncytin-2* using the two chimeras. As a result, their expression levels were decreased by the SPRE mutation, and the effects were smaller than WT-*syncytin-2* (Fig. 6b). Therefore, SPRE dependency is thought to be determined by the entire coding sequence such as several motifs and/or structural interactions rather than a particular motif. These data suggest that SPRE supports the efficient expression of ERV-derived genes whose expression was suppressed.

## Discussion

In this study, we characterized a functional retroviral RNA element, termed SPRE, found in a variety of distinct ERV families, including the ERV-derived *syncytin* genes. In particular, a 17-nt (5′-TCAGCAGGAAGCAGTTA-3′) was identified as the functional core motif of this element (Fig. 1). The discovery of the SPRE-core motif enabled sequence-based searches and revealed that 378 ERV families harbor the SPRE-like elements consisting of the SPRE-core motif and two upstream and downstream C-rich motifs (Fig. 2d). Although SPRE-binding proteins are still unclear, the loss of SPRE activity by the mutagenesis in each motif suggests that the SPRE activity requires multiple proteins that bind to each motif and the formation of an “SPRE-proteins complex.” The RNA secondary structures are also generally crucial for the function of RNA elements. However, our predictions of RNA secondary structures of SPRE-like elements in *syncytin* genes varied markedly (Additional file 4: Fig. S4). The specific secondary structures may not contribute to the function of SPRE, or common structures may be formed by protein binding *in vivo* that cannot be revealed by predictions based on the nucleotide sequences alone.

We found SPRE-like elements in the genomes of a variety of mammalian species (Fig. 3). While SPRE-like elements were detected in all major mammalian clades, their copy numbers varied markedly among species. As most SPRE-like elements were found in ERVs (LTR retrotransposons) (Fig. 2b), the increase in copy numbers of SPRE is mainly due to retrotransposition of ERVs and/or horizontal transmission by ancient infection. Considering the repeated invasion of a wide variety of mammalian lineages, the ancient SPRE-harboring retroviruses may have been a prospering viral group(s). It should be noted that the “SPRE-harboring retroviruses” do not indicate a single viral clade, as SPRE-like elements were identified not only from the ERV1 group but also from the ERV2 group (Additional file 6: Table S1). SPRE-harboring *syncytin* genes also did not form a single clade in a phylogenetic tree of Syncytin and exogenous retroviral Env proteins (Additional file 3: Fig. S3). These data suggest that the SPRE-like elements emerged in a convergent manner during evolution or were lost in some specific lineage. Previous attempts at systematic classification of human ERVs revealed their mosaic structures between families and even distinct classes due to recombination [29]. Therefore, it is possible that the SPRE-like elements were inherited from one retrovirus to another retrovirus through recombination.

SPRE has not been identified among the current prevailing exogenous retroviruses. Since SPRE is functional in mammalian and avian cells (Fig. 5f), It has the potential to provide a functional advantage to exogenous retroviruses infecting a wide variety of host species. Therefore, the reason why SPRE is not present in exogenous retroviruses is an enigma. One possibility is that the spread of SPREs was not caused mainly by the horizontal transmission of exogenous retroviruses but by vertical transmission with recombination of ERVs in each host genome. Another possibility is simply due to incomplete sampling of known exogenous retroviruses. Current exogenous retroviruses are 68 species according to International Committee on Taxonomy of Viruses 2020 release (https://talk.ictvonline.org/). The proportion of LTR-type retrotransposons with SPRE is roughly calculated to be 0.93% (364/38,964 families) in all LTR-type retrotransposons in Dfam 3.3 (Fig. 2). Thus, the number of known exogenous retroviruses may be too small to include SPRE-harboring retroviruses.

The SPRE mutation increased the length of poly(A) tails in *syncytin-1* mRNA (Fig. 4g). The relationship between the poly(A) tail sizes and translation efficiency has not been observed or only observed in limited developmental stages in early comprehensive studies on the poly(A) tail length [44, 45]. Further, a more recent study revealed that a short poly(A) tail is a feature of highly translated genes [43, 46]. Although this phenomenon was not observed in *syncytin-2* mRNA, the longer poly(A) tail of *syncytin-1* mRNA would be one of the mechanisms underlying its reduced translation efficiency.

SPRE promoted gene expression of HIV-1-*gag* and MLV-*gag* but not of SFV-*gag*. If there are common features among SPRE-responsive genes, they may reflect differences in the post-transcriptional regulatory strategies of various retroviruses. An important clue was obtained from experiments with codon-optimized syncytin-2 (Fig. 6). Codon-optimized *syncytin-2* induced cell fusion despite the mutation into SPRE, suggesting that the expression of wild-type *syncytin-2* is restricted in a coding sequence-dependent manner, and SPRE counteracts the repressive regulation. The same phenomenon is observed in HIV-1, whose mRNAs are regulated to be inadequately expressed without Rev/RRE. One explanation for this is that their codon frequencies are inefficient. HIV genes, *gag*, *pol*, and *env*, have different codon usages from highly expressing host genes, and codon-optimization makes their expression Rev/RRE-independent [47, 48]. We first hypothesized that SPRE-harboring *syncytin* genes have different codon frequencies from other *syncytin* genes but could not observe specific associations between SPRE-dependency and codon usages (Additional file 5: Fig. S5). In HIV-1 *gag* and *pol* coding sequences, multiple negatively acting sequences were detected by serial clustered mutagenesis [49]. Similarly, we cannot rule out the possibility that some repressive RNA motifs in the *syncytin-2* coding sequence were removed by codon-optimization. In either case, these findings revealed the convergent evolution of complex viral RNA regulation between current and ancient retroviruses, in which other RNA regulatory systems counteract the repressive regulation intrinsic to viral coding sequences.

The co-option of retroviral regulatory elements is also crucial for the regulation of host genes. LTRs of ERVs contain various *cis*-acting elements, enhancers, promoters, and polyadenylation signals, that influence host gene expression, contributing to the diversification of species-specific gene expression in immune systems [50] and placental development [51, 52]. By analogy, it is also possible that RNA elements in ERVs and other retrotransposons contribute to the host gene regulatory networks. Totals of 27.7% of mouse and 28.5% of human RefSeq transcripts contain at least one retrotransposon in their 3′ UTR, and percentages of ERVs and retrotransposons in 3′ UTRs negatively correlate with gene expression levels [53]. Although the molecular mechanisms of this phenomenon are still unclear, the SPRE findings suggest that ERV-derived elements in 3′ UTRs may affect host gene expression via viral RNA motifs. ERV-derived RNAs also function as long non-coding RNAs (lncRNAs). Recent studies reported that ERV-derived lncRNAs can affect the host gene regulatory networks by binding to host RNA binding proteins [54–56]. Such lncRNAs may harbor RNA elements that have evolved to provide advantages to ancient retroviruses. Therefore, in addition to characterizing transcripts of ERV-derived genes such as *syncytin* genes, functional analysis of ERV-derived lncRNAs may also provide the opportunity to discover more hidden RNA elements and reveal further insights into the evolution of retroviral RNA elements.

## Conclusions

We characterized a retroviral RNA element found in distinct ERV families and ERV-derived *syncytin* genes. SPRE-like elements were identified in a variety of mammalian genomes and their copy numbers varied markedly among species while the elements have not been identified among the current prevailing exogenous retroviruses. Enhancement of gene expression by SPRE depended on the reporter genes, and codon-optimized *syncytin-2* showed SPRE-independent expression different from wild-type *syncytin-2*. Exploring RNA elements in ERVs provides opportunities to investigate the post-transcriptional regulatory mechanisms of extinct retroviruses and shed light on new aspects of the long-term evolution of RNA elements in retroviruses hidden in our genomes.

## Methods

### Cell cultures

293T (#RCB2202, Riken BioResource Research Center, Tsukuba, Japan), COS-7 (#CRL-1651, ATCC, Manassas, VA, USA), NIH3T3-3-4 (#RCB1862, Riken BioResource Research Center), MDCK (#CCL-34, ATCC), and QT6 (#CRL-1708, ATCC) were cultured in Dulbecco’s modified Eagle’s medium (#D5796, Sigma Aldrich) supplemented with 10% heat-inactivated fetal calf serum (#10,270,106, Thermo Fisher Scientific, Waltham, MA, USA), and penicillin (100 units/mL) and streptomycin (100 mg/mL) (#09367-34, Nacalai Tesque, Kyoto, Japan) at 37 °C in a humidified atmosphere of 5% CO_2_ in air.

### Plasmids

Syncytin-1 and Syncytin-2 expression plasmids of the phCMV3 backbone (#P003300, Genlantis, San Diego, CA, USA) were constructed previously [21]. For minigene construction, ERVWE1 and ERVFRDE1 loci were inserted into the SmaI site of pSP73 (#P2221, Promega, Madison, WI, USA) with the fragments of cytomegalovirus (CMV) enhancer and promoter amplified from pcDNA3.1 (#V79020, Thermo Fisher Scientific) using NEBuilder HiFi DNA Assembly Master Mix (#M5520AA, New England Biolabs, Ipswich, MA, USA). For the site-directed mutagenesis, linearized vectors with mutations were generated by inverse PCR, and they were joined and cyclized by NEBuilder. For the construction of HiBiT reporter plasmids, HIV-1 Gag from pHG [13] was amplified by PCR and inserted into the EcoRI and BamHI sites of phCMV3, and the HiBiT sequence was inserted into the C-terminus by inverse PCR followed by NEBuilder. HIV-1 *gag* was replaced with MLV *gag* amplified from pGag-Pol-IRES-bsr [57] and SFV *gag* amplified from pJM356 [58]. For the NanoLuc expression plasmid, a fragment of the NanoLuc gene was amplified from pNL1.1 (#N1001, Promega) and inserted into the HindIII and EcoRI sites of phCMV3. SPRE-like elements were synthesized and inserted between the EcoRI and NotI sites of each HiBiT reporter plasmid. For the construction of codon-optimized *syncytin-2* minigenes, dsDNA coding codon-optimized *syncytin-2* was synthesized by Eurofins Genomics (Tokyo, Japan). Synthesized DNA was amplified and replaced with the *syncytin-2* coding sequence in pFRDE1. All PCRs described above were carried out using KOD One Master Mix (#KMM-101, TOYOBO, Osaka, Japan) with a C1000 Touch thermal cycler (Bio-Rad, Hercules, CA, USA). Primer sequences are listed in Additional file 10: Table S5.

### Fusion-dependent luciferase assay

A fusion-dependent luciferase assay was performed as described previously [59] with some modifications. 293T cells were seeded onto 24-well plates (2.5 × 10^5^ cells/well). On the next day, cells were co-transfected with each Syncytin expression plasmid (500 ng), pT7EMCLuc (500 ng), and pRL-TK (50 ng) with 0.7 µl of Avalanche Everyday Transfection Reagent (#EZT-EVDY-1, EZ Biosystems, College Park, MD, USA) according to the manufacturer’s instructions. Six hours post-transfection, cells were resuspended and cocultured with 293T cells transfected with a T7 polymerase expression vector, pCAGT7. Then, 24 h after coculture, luciferase activities of cell lysates were measured using the Dual-Luciferase Reporter Assay System (#E1910, Promega). All experiments were conducted more than three times independently.

### HiBiT luciferase assay

293T, COS-7, NIH3T3-3-4, MDCK, and QT6 cells seeded in 24-well plates (1.2–2.5 × 10^5^ cells/well) were transfected with HiBiT reporter plasmid (500 ng) with Avalanche Everyday Transfection Reagent. Twenty-four hours after transfection, cells were subjected to the luciferase assay using Nano Glo HiBiT Lytic Detection System (#N3030, Promega).

### Northern blot analysis

For sample preparation, two µg of each minigene plasmid was used to transfect 293T cells seeded in 6-well plates (1 × 10^6^ cells/well). Twenty-four hours after transfection, total RNA was extracted using RNAzol RT (#RN109, Molecular Research Center, Cincinnati, OH, USA) and stored at − 80 °C. The protocol and reagents used for Northern blotting were followed by the manufacturer’s protocol, “DIG application manual for Filter Hybridization” (Roche, Basel, Switzerland). Briefly, for probe construction, DNA fragments with the T7 promoter were obtained by PCR with the T7 promoter sequence-attached primers listed in Additional file 10: Table S5. The amplicons were used as templates for in vitro transcription using DIG RNA Labeling Kit (#11175025910, Roche). RNA samples were mixed with 2 × Loading Buffer and heated at 65 °C for 10 min. Then, Each RNA sample (2.5 µg/lane) was loaded into 1.5% agarose gel in 1 × morpholine propane sulfonic acid buffer containing 2% formaldehyde. After electrophoresis at 100 V for 180 min, samples were transferred onto a positively charged nylon membrane (#11209272001, Roche) overnight and fixed by UV cross-linking. DIG-labeled RNA probes were hybridized with DIG Easy Hyb (#11796895001, Roche) at 68 °C overnight, and anti-Digoxigenin-AP, Fab fragments from sheep (#11093274910, Roche) were bound to probes with Blocking Reagent (#11096176001, Roche). Signal detection was performed by CDP-Star (#11685627001, Roche) with LAS-4000 (Fujifilm, Tokyo, Japan).

### Poly(A) tail-targeted PCR

Total RNA was isolated using RNAzol RT from 293T cells 24 h after transfection. Universal miRNA Cloning Linker (#S1315S, New England Biolabs) was linked to the 3′-terminus of RNA using T4 RNA Ligase 2, truncated K227Q (#M0351S, New England Biolabs). Then, cDNA was synthesized using Verso cDNA Synthesis Kit (#AB1453, Thermo Fisher Scientific), and PCR was performed using KOD One PCR Master Mix (#KMM-101, TOYOBO). The resulting PCR fragments were cloned into pSP73 and verified by Sanger sequencing. Primers are listed in Additional file 10: Table S5.

### Sequence search for SPRE-like elements in the Dfam database

Nucleotide sequences of 273,655 repetitive DNA families were retrieved from an EMBL file of Dfam release 3.3 (April/2021) (https://www.dfam.org/home) [23]. For the first step of the search, the families with a complete sequence match to the 17-nt SPRE-core motif (5′-TCAGCAGGAAGCAGTTA-3′) were extracted. Then, the SPRE-core motifs with their 5′ and 3′ flanking 40-nt sequences (total 97-nt) were retrieved from the 22 families and aligned using MAFFT version b7.402 [60]. The alignment was subjected to an HMM-based search in the 273,655 families of Dfam entries using the nhmmer program of HMMER vertion 3.3.1 [24] for the second-step search. The alignment of the resultant SPRE-like elements was output using nhmmer with “-A -E 1E-5” options and used for the re-search using nhmmer. The second step was repeated 10 times, and the number of hits peaked in the fourth cycle. The alignment of 393 hits from 378 families was visualized by WebLogo3 [61].

### Sequence search for the SPRE-like elements in viral sequences and host genomes

To search for SPRE-like elements in host genomes, genomic sequences of 422 species of mammals and 499 species of birds available in the NCBI genome database as of December 13th, 2020 (https://www.ncbi.nlm.nih.gov/genome/) were retrieved. To search for SPRE-like elements in exogenous viruses, all viral sequences in the NCBI nucleotide collection, including partial viral sequences, were retrieved from NCBI virus with taxonomy ID:10239 (https://www.ncbi.nlm.nih.gov/labs/virus/) on October 13th, 2020. Nhmmer was used for the above sequence search with the HMM profile constructed from the SPRE search in the Dfam database with “--tblout -E 1E-5” options.

### Analysis of the overlap of the SPRE-like elements with RepeatMasker tracks

Table output files of nhmmer were converted to BED files by custom scripts. Output files of RepeatMasker (http://www.repeatmasker.org/) were retrieved from the FTP directory for RefSeq assemblies in the NCBI website (https://www.ncbi.nlm.nih.gov/assembly). RepeatMasker version, referenced repetitive DNA databases, and the links to raw output files of RepeatMasker are listed in Additional file 9: Table S4. RepeatMasker tracks that overlapped with the SPRE-like elements were detected by BEDtools intersect command with “-s -wa -wb” options [62].

### Construction of a phylogenetic tree

The MEGA X software suite [63] was used for phylogenetic analyses as follows. Since the transmembrane (TM) domain of *Env* proteins is known to be more conserved than the surface (SU) domain in many retroviruses [64], the amino acid sequences of the TM domain were used for phylogenetic analysis. The amino acid sequences were aligned by the MUSCLE program with default options. The maximum likelihood tree was generated with the WAG+F model as suggested by the MEGA X model selection. The robustness of the phylogenetic tree was evaluated by 1000 bootstrap duplicate data sets.

### Analysis of codon usages

Relative codon frequencies of *syncytin* genes and reporter genes were calculated using Codon Usage Generator v2.4 [65, 66]. A heatmap was generated using the heatmap.2 function of R version 4.1.0.

## Supplementary Information

**Additional file 1: Fig. S1.** Alignment of SPRE-core motifs with 40-nt flanking from 22 Dfam families obtained by first-step search. The alignment was visualized by AliView version1.27 [67].

**Additional file 2: Fig. S2.** Numbers of hits including all Dfam families (n = 273,655) in second-step search.

**Additional file 3: Fig. S3.** Maximum likelihood phylogenetic tree of TM domains in Syncytin proteins and other retroviral Env proteins. Bootstrap support values are displayed at the nodes. SPRE-harboring Syncytins are indicated in red. Accession numbers of sequences are listed in Additional file 11: Table S6.

**Additional file 4: Fig. S4.** RNA secondary structures of SPRE-like elements. Structure predictions were conducted by Sfold webserver (http://sfold.wadsworth.org/cgi-bin/srna.pl) with default parameters.

**Additional file 5: Fig. S5.** Heatmap classification of *syncytin* and reporter genes by their codon frequencies.

**Additional file 6: Table S1.** Dram families harboring SPRE-like elements and their annotations.

**Additional file 7: Table S2.** The numbers of SPRE-like elements in mammalian genomes.

**Additional file 8: Table S3.** The numbers of SPRE-like elements in avian genomes.

**Additional file 9: Table S4.** RepeatMasker versions and referenced databases.

**Additional file 10: Table S5.** Primers used in this study.

**Additional file 11: Table S6.** Accession numbers used for phylogenetic tree of syncytin genes.

## Data Availability

All relevant data are contained in the manuscript or Additional files.

## Abbreviations

ERV: Endogenous retrovirus
HIV: Human immunodeficiency virus
MPMV: Mason-Pfizer monkey virus
MLV: Murine leukemia virus
SFV: Simian foamy virus
CMV: Cytomegalovirus
RRE: Rev-responsive element
CTE: Constitutive transport element
CAE: Cytoplasmic accumulation element
SPRE: Syncytin post-transcriptional regulatory element
UTR: Untranslated region
HMM: Hidden Markov model

## References

[1] Lavialle C, Cornelis G, Dupressoir A, Esnault C, Heidmann O, Vernochet C (2013). Paleovirology of ‘*syncytins*’, retroviral *env* genes exapted for a role in placentation. Philos Trans R Soc B Biol Sci.

[2] Chuong EB, Elde NC, Feschotte C (2017). Regulatory activities of transposable elements: from conflicts to benefits. Nat Rev Genet.

[3] Foroushani AK, Chim B, Wong M, Rastegar A, Smith PT, Wang S (2020). Posttranscriptional regulation of human endogenous retroviruses by RNA-binding motif protein 4, RBM4. Proc Natl Acad Sci USA.

[4] Daly TJ, Cook KS, Gray GS, Maione TE, Rusche JR (1989). Specific binding of HIV-1 recombinant Rev protein to the Rev-responsive element in vitro. Nature.

[5] Zapp ML, Green MR (1989). Sequence-specific RNA binding by the HIV-1 Rev protein. Nature.

[6] Fornerod M, Ohno M, Yoshida M, Mattaj IW (1997). CRM1 is an export receptor for leucine-rich nuclear export signals. Cell.

[7] Arrigo SJ, Chen ISY (1991). Rev is necessary for translation but not cytoplasmic accumulation of HIV-1 vif, vpr, and env/vpu 2 RNAs. Genes Dev.

[8] Hanly SM, Rimsky LT, Malim MH, Kim JH, Hauber J, Duc Dodon M (1989). Comparative analysis of the HTLV-I Rex and HIV-1 Rev trans-regulatory proteins and their RNA response elements. Genes Dev.

[9] Indik S, Günzburg WH, Salmons B, Rouault F (2005). A novel, mouse mammary tumor virus encoded protein with Rev-like properties. Virology.

[10] Bray M, Prasad S, Dubay JW, Hunter E, Jeang KT, Rekosh D (1994). A small element from the Mason-Pfizer monkey virus genome makes human immunodeficiency virus type 1 expression and replication Rev-independent. Proc Natl Acad Sci USA.

[11] Grüter P, Tabernero C, Von Kobbe C, Schmitt C, Saavedra C, Bachi A (1998). TAP, the human homolog of Mex67p, mediates CTE-dependent RNA export from the nucleus. Mol Cell.

[12] Jin L, Guzik BW, Bor YC, Rekosh D, Hammarskjöld ML (2003). Tap and NXT promote translation of unspliced mRNA. Genes Dev.

[13] Sakuma T, Davila JI, Malcolm JA, Kocher J-PA, Tonne JM, Ikeda Y (2014). Murine leukemia virus uses NXF1 for nuclear export of spliced and unspliced viral transcripts. J Virol.

[14] Takata M, Soll SJ, Emery A, Blanco-Melo D, Swanstrom R, Bieniasz PD (2018). Global synonymous mutagenesis identifies cis-acting RNA elements that regulate HIV-1 splicing and replication. PLoS Pathog.

[15] Villesen P, Aagaard L, Wiuf C, Pedersen FS (2004). Identification of endogenous retroviral reading frames in the human genome. Retrovirology.

[16] Wildschutte JH, Williams ZH, Montesion M, Subramanian RP, Kidd JM, Coffin JM (2016). Discovery of unfixed endogenous retrovirus insertions in diverse human populations. Proc Natl Acad Sci USA.

[17] Magin C, Löwer R, Löwer J (1999). cORF and RcRE, the Rev/Rex and RRE/RxRE homologues of the human endogenous retrovirus family HTDV/HERV-K. J Virol.

[18] Yang J, Bogerd HP, Peng S, Wiegand H, Truant R, Cullen BR (1999). An ancient family of human endogenous retroviruses encodes a functional homolog of the HIV-1 Rev protein. Proc Natl Acad Sci USA.

[19] Mi S, Lee X, Li X, Veldman GM, Finnerty H, Racie L (2000). Syncytin is a captive retroviral envelope protein involved in human placental morphogenesis. Nature.

[20] Blond J-L, Lavillette D, Cheynet V, Bouton O, Oriol G, Chapel-Fernandes S (2000). An envelope glycoprotein of the human endogenous retrovirus HERV-W is expressed in the human placenta and fuses cells expressing the type D mammalian retrovirus receptor. J Virol.

[21] Kitao K, Tanikaga T, Miyazawa T (2019). Identification of a post-transcriptional regulatory element in the human endogenous retroviral syncytin-1. J Gen Virol.

[22] Blaise S, de Parseval N, Benit L, Heidmann T (2003). Genomewide screening for fusogenic human endogenous retrovirus envelopes identifies syncytin 2, a gene conserved on primate evolution. Proc Natl Acad Sci USA.

[23] Storer J, Hubley R, Rosen J, Wheeler TJ, Smit AF (2021). The Dfam community resource of transposable element families, sequence models, and genome annotations. Mob DNA.

[24] Wheeler TJ, Eddy SR (2013). nhmmer: DNA homology search with profile HMMs. Bioinformatics.

[25] Laufer G, Mayer J, Mueller BF, Mueller-Lantzsch N, Ruprecht K (2009). Analysis of transcribed human endogenous retrovirus W env loci clarifies the origin of multiple sclerosis-associated retrovirus env sequences. Retrovirology.

[26] Kryukov K, Imanishi T (2016). Human contamination in public genome assemblies. PLoS ONE.

[27] Cantrell MA, Ederer MM, Erickson IK, Swier VJ, Baker RJ, Wichman HA (2005). MysTR: an endogenous retrovirus family in mammals that is undergoing recent amplifications to unprecedented copy numbers. J Virol.

[28] Grandi N, Cadeddu M, Blomberg J, Tramontano E (2016). Contribution of type W human endogenous retroviruses to the human genome: characterization of HERV-W proviral insertions and processed pseudogenes. Retrovirology.

[29] Vargiu L, Rodriguez-Tomé P, Sperber GO, Cadeddu M, Grandi N, Blikstad V (2016). Classification and characterization of human endogenous retroviruses mosaic forms are common. Retrovirology.

[30] Grandi N, Cadeddu M, Blomberg J, Mayer J, Tramontano E (2018). HERV-W group evolutionary history in non-human primates: characterization of ERV-W orthologs in Catarrhini and related ERV groups in Platyrrhini. BMC Evol Biol.

[31] Grandi N, Pisano MP, Demurtas M, Blomberg J, Magiorkinis G, Mayer J (2020). Identification and characterization of ERV-W-like sequences in Platyrrhini species provides new insights into the evolutionary history of ERV-W in primates. Mob DNA.

[32] Imakawa K, Nakagawa S (2017). The phylogeny of placental evolution through dynamic integrations of retrotransposons. Prog Mol Biol Transl Sci.

[33] Imakawa K, Nakagawa S, Miyazawa T (2015). Baton pass hypothesis: successive incorporation of unconserved endogenous retroviral genes for placentation during mammalian evolution. Genes Cells.

[34] Esnault C, Cornelis G, Heidmann O, Heidmann T (2013). Differential evolutionary fate of an ancestral primate endogenous retrovirus envelope gene, the EnvV Syncytin, captured for a function in placentation. PLoS Genet.

[35] Dupressoir A, Marceau G, Vernochet C, Benit L, Kanellopoulos C, Sapin V (2005). Syncytin-A and syncytin-B, two fusogenic placenta-specific murine envelope genes of retroviral origin conserved in Muridae. Proc Natl Acad Sci USA.

[36] Redelsperger F, Cornelis G, Vernochet C, Tennant BC, Catzeflis F, Mulot B (2014). Capture of syncytin-Mar1, a fusogenic endogenous retroviral envelope gene involved in placentation in the rodentia squirrel-related clade. J Virol.

[37] Heidmann O, Vernochet C, Dupressoir A, Heidmann T (2009). Identification of an endogenous retroviral envelope gene with fusogenic activity and placenta-specific expression in the rabbit: a new “syncytin” in a third order of mammals. Retrovirology.

[38] Cornelis G, Heidmann O, Degrelle SA, Vernochet C, Lavialle C, Letzelter C (2013). Captured retroviral envelope syncytin gene associated with the unique placental structure of higher ruminants. Proc Natl Acad Sci USA.

[39] Nakaya Y, Koshi K, Nakagawa S, Hashizume K, Miyazawa T (2013). Fematrin-1 is involved in fetomaternal cell-to-cell fusion in Bovinae placenta and has contributed to diversity of ruminant placentation. J Virol.

[40] Cornelis G, Heidmann O, Bernard-Stoecklin S, Reynaud K, Veron G, Mulot B (2012). Ancestral capture of syncytin-Car1, a fusogenic endogenous retroviral envelope gene involved in placentation and conserved in Carnivora. Proc Natl Acad Sci USA.

[41] Cornelis G, Vernochet C, Malicorne S, Souquere S, Tzika AC, Goodman SM (2014). Retroviral envelope syncytin capture in an ancestrally diverged mammalian clade for placentation in the primitive Afrotherian tenrecs. Proc Natl Acad Sci USA.

[42] Cornelis G, Vernochet C, Carradec Q, Souquere S, Mulot B, Catzeflis F (2015). Retroviral envelope gene captures and syncytin exaptation for placentation in marsupials. Proc Natl Acad Sci USA.

[43] Lima SA, Chipman LB, Nicholson AL, Chen YH, Yee BA, Yeo GW (2017). Short poly(A) tails are a conserved feature of highly expressed genes. Nat Struct Mol Biol.

[44] Chang H, Lim J, Ha M, Kim VN (2014). TAIL-seq: Genome-wide determination of poly(A) tail length and 3’ end modifications. Mol Cell.

[45] Subtelny AO, Eichhorn SW, Chen GR, Sive H, Bartel DP (2014). Poly(A)-tail profiling reveals an embryonic switch in translational control. Nature.

[46] Nicholson AL, Pasquinelli AE (2019). Tales of detailed Poly(A) Ttails. Trends Cell Biol.

[47] Haas J, Park EC, Seed B (1996). Codon usage limitation in the expression of HIV-1 envelope glycoprotein. Curr Biol.

[48] Kotsopoulou E, Kim VN, Kingsman AJ, Kingsman SM, Mitrophanous KA (2000). A Rev-independent human immunodeficiency virus type 1 (HIV-1)-based vector that exploits a codon-optimized HIV-1 gag-pol gene. J Virol.

[49] Schneider R, Campbell M, Nasioulas G, Felber BK, Pavlakis GN (1997). Inactivation of the human immunodeficiency virus type 1 inhibitory elements allows Rev-independent expression of Gag and Gag/protease and particle formation. J Virol.

[50] Chuong EB, Elde NC, Feschotte C (2016). Regulatory evolution of innate immunity through co-option of endogenous retroviruses. Science.

[51] Chuong EB, Rumi MAK, Soares MJ, Baker JC (2013). Endogenous retroviruses function as species-specific enhancer elements in the placenta. Nat Genet.

[52] Dunn-Fletcher CE, Muglia LM, Pavlicev M, Wolf G, Sun MA, Hu YC (2018). Anthropoid primate–specific retroviral element THE1B controls expression of CRH in placenta and alters gestation length. PLoS Biol.

[53] Faulkner GJ, Kimura Y, Daub CO, Wani S, Plessy C, Irvine KM (2009). The regulated retrotransposon transcriptome of mammalian cells. Nat Genet.

[54] Jin X, Xu XE, Jiang YZ, Liu YR, Sun W, Guo YJ (2019). The endogenous retrovirus-derived long noncoding RNA TROJAN promotes triple-negative breast cancer progression via ZMYND8 degradation. Sci Adv.

[55] Zhou B, Qi F, Wu F, Nie H, Song Y, Shao L (2019). Endogenous retrovirus-derived long noncoding RNA enhances innate immune responses via derepressing RELA expression. MBio.

[56] Wilson KD, Ameen M, Guo H, Abilez OJ, Tian L, Mumbach MR (2020). Endogenous retrovirus-derived lncRNA BANCR promotes cardiomyocyte migration in humans and non-human primates. Dev Cell.

[57] Morita S, Kojima T, Kitamura T (2000). Plat-E: an efficient and stable system for transient packaging of retroviruses. Gene Ther.

[58] Yoshikawa R, Nakagawa S, Okamoto M, Miyazawa T (2014). Construction of an infectious clone of simian foamy virus of Japanese macaque (SFVjm) and phylogenetic analyses of SFVjm isolates. Gene.

[59] Suenaga T, Satoh T, Somboonthum P, Kawaguchi Y, Mori Y, Arase H (2010). Myelin-associated glycoprotein mediates membrane fusion and entry of neurotropic herpesviruses. Proc Natl Acad Sci USA.

[60] Katoh K, Standley DM (2013). MAFFT multiple sequence alignment software version 7: improvements in performance and usability. Mol Biol Evol.

[61] Crooks GE, Hon G, Chandonia J-M, Brenner SE (2004). WebLogo: a sequence logo generator. Genome Res.

[62] Quinlan AR, Hall IM (2010). BEDTools: a flexible suite of utilities for comparing genomic features. Bioinformatics.

[63] Kumar S, Stecher G, Li M, Knyaz C, Tamura K (2018). MEGA X: Molecular evolutionary genetics analysis across computing platforms. Mol Biol Evol.

[64] Bénit L, Dessen P, Heidmann T (2001). Identification, phylogeny, and evolution of retroviral elements based on their envelope genes. J Virol.

[65] Kanaya S, Yamada Y, Kudo Y, Ikemura T (1999). Studies of codon usage and tRNA genes of 18 unicellular organisms and quantification of *Bacillus subtilis* tRNAs: Gene expression level and species-specific diversity of codon usage based on multivariate analysis. Gene.

[66] Suzuki H, Brown CJ, Forney LJ, Top EM (2008). Comparison of correspondence analysis methods for synonymous codon usage in bacteria. DNA Res.

[67] Larsson A (2014). AliView: a fast and lightweight alignment viewer and editor for large datasets. Bioinformatics.

