# Supplementary figures and images for: An ancient retroviral RNA element hidden in mammalian genomes and its involvement in co-opted retroviral gene regulation
Source: Retrovirology. 2021 Nov 10;18:36. doi: 10.1186/s12977-021-00580-2 (PMC8579622; doi:10.1186/s12977-021-00580-2)

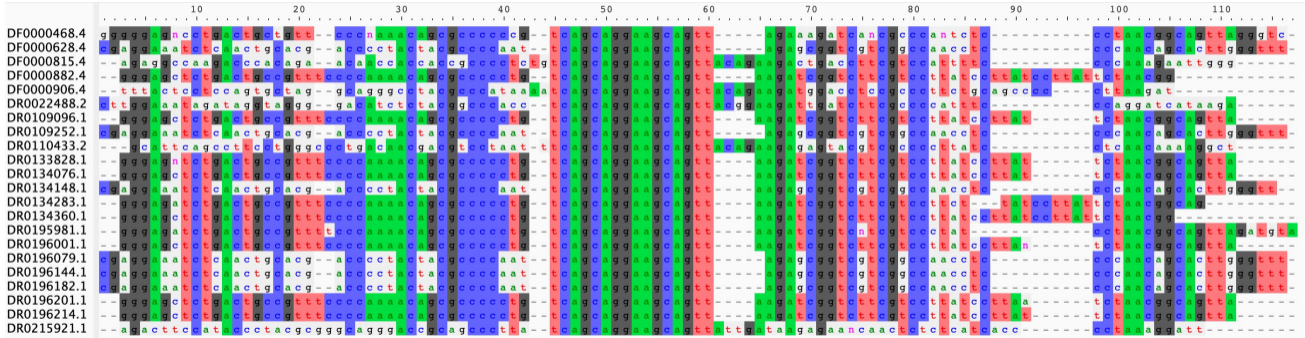

Supplement: Supplementary file 1 — Additional file 1: Fig. S1. Alignment of SPRE-core motifs with 40-nt flanking from 22 Dfam families obtained by first-step search. The alignment was visualized by AliView version1.27 [67]. [file 12977_2021_580_MOESM1_ESM.pdf]

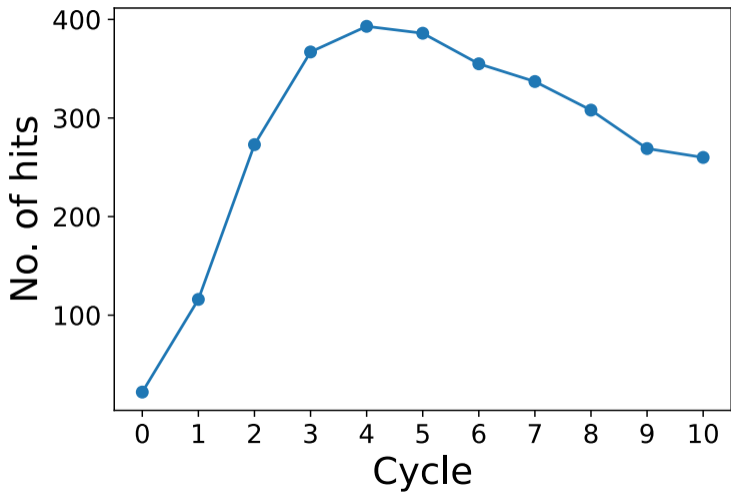

Supplement: Supplementary file 2 — Additional file 2: Fig. S2. Numbers of hits including all Dfam families (n = 273,655) in second-step search. [file 12977_2021_580_MOESM2_ESM.pdf]

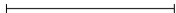

Supplement: Supplementary file 3 — Additional file 3: Fig. S3. Maximum likelihood phylogenetic tree of TM domains in Syncytin proteins and other retroviral Env proteins. Bootstrap support values are displayed at the nodes. SPRE-harboring Syncytins are indicated in red. Accession numbers of sequences are listed in Additional file 11: Table S6. [file 12977_2021_580_MOESM3_ESM.pdf]

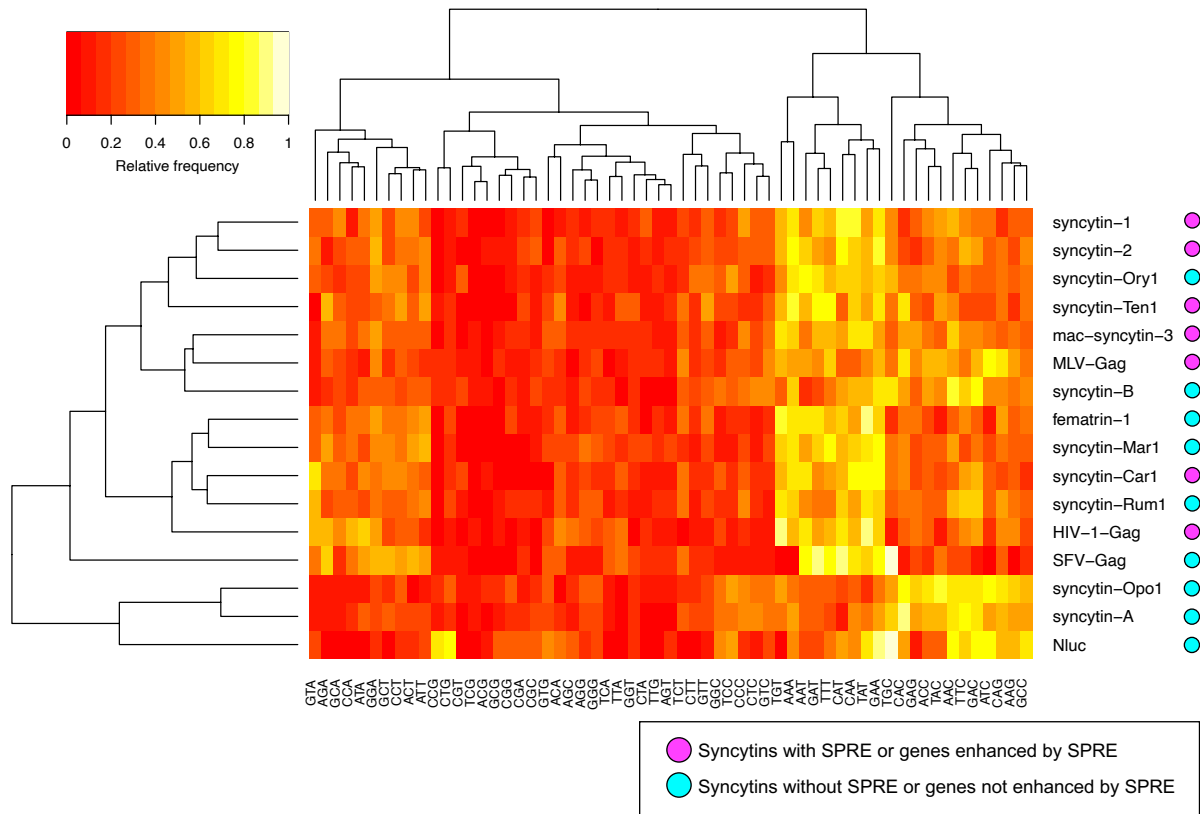

Supplement: Supplementary file 5 — Additional file 5: Fig. S5. Heatmap classification of syncytin and reporter genes by their codon frequencies. [file 12977_2021_580_MOESM5_ESM.pdf]
