# Supplementary material for: An ancient retroviral RNA element hidden in mammalian genomes and its involvement in co-opted retroviral gene regulation
Source: Retrovirology. 2021 Nov 10;18:36. doi: 10.1186/s12977-021-00580-2 (PMC8579622; doi:10.1186/s12977-021-00580-2)
Supplement: Supplementary file 4 — Additional file 4: Fig. S4. RNA secondary structures of SPRE-like elements. Structure predictions were conducted by Sfold webserver (http://sfold.wadsworth.org/cgi-bin/srna.pl) with default parameters. [file 12977_2021_580_MOESM4_ESM.pdf]

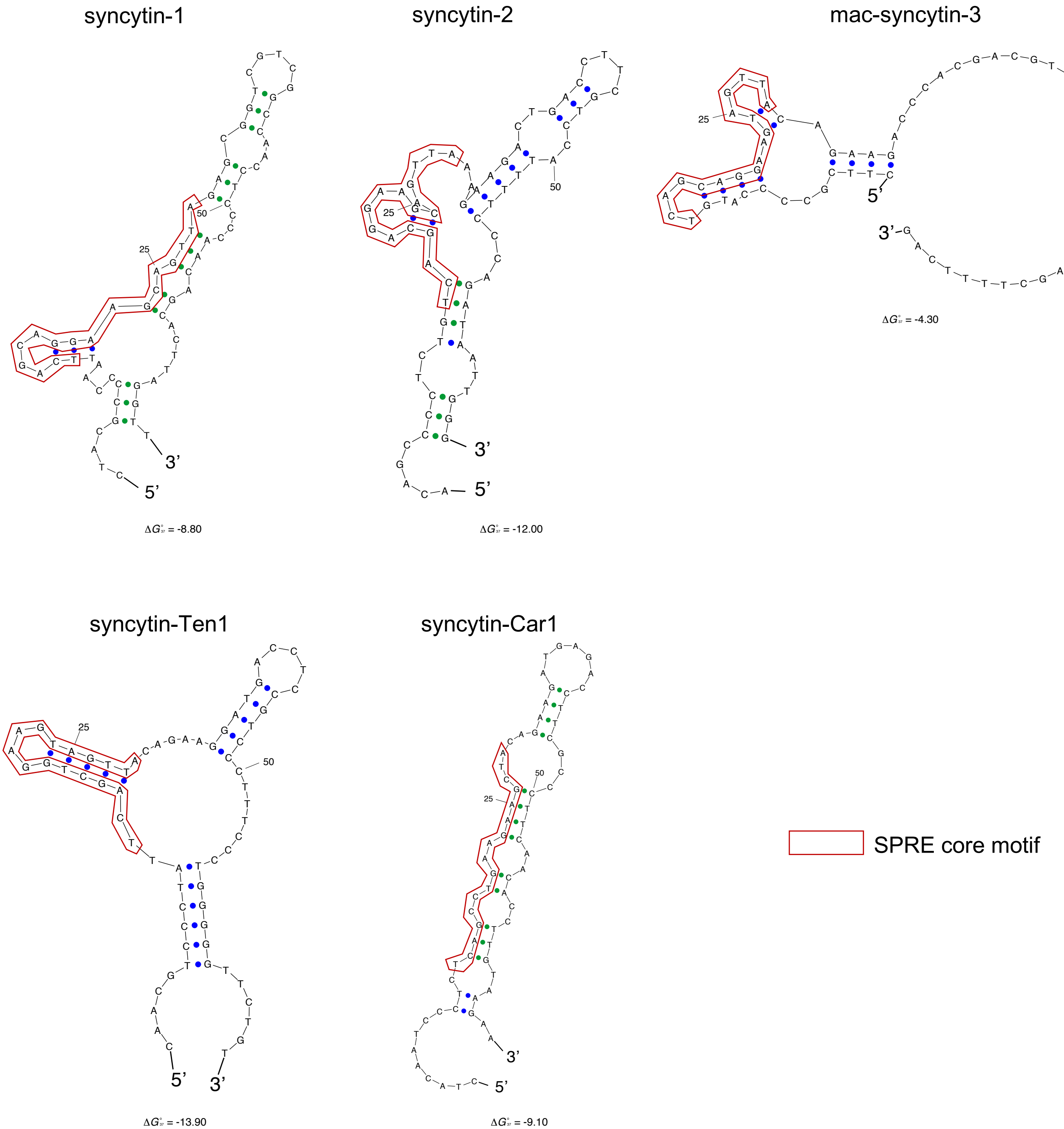
syncytin-1
syncytin-2
mac-syncytin-3
ΔG°37 = -8.80
ΔG°37 = -12.00
ΔG°37 = -4.30
syncytin-Ten1
syncytin-Car1
SPRE core motif
ΔG°37 = -13.90
ΔG°37 = -9.10
